# The mediating effect of immune-inflammatory indices in shift work and hypertension: a cohort study in China

**DOI:** 10.5271/sjweh.4287

**Published:** 2026-05-01

**Authors:** Haixia Lu, Yuxin Jin, Changxue Huang, Lan Lin, Xiangran Zhang, Wanyu Wang, Yulong Lian

**Affiliations:** 1 Division of Epidemiology and Medical Statistics, School of Public Health, Nantong University, Nantong, Jiangsu, China.; # Contributed equally to this paper.

**Keywords:** immune-inflammation index, inflammatory marker

## Abstract

**Objectives:**

Global hypertension prevalence is increasing. Studies of the association of shift work with hypertension have produced inconsistent results, and the role of immune-inflammatory indices in this link remains unclear. This study aimed to clarify the association of shift work with hypertension and to identify the mediating effects of immune-inflammatory indices on hypertension.

**Method:**

This cohort study assessed 1322 oil workers, with baseline data collected from 2013−2015 and final follow-up in 2023. The definition of shift work was in accordance with the International Labor Organization’s Convention on Night Work (No. 171). Hypertension was diagnosed according to the Chinese Hypertension Prevention and Treatment Guidelines (2018 revised edition). Smooth curve fitting, piecewise regression modeling, and the Karlson-Holm-Breen method were used to analyze the associations among immune-inflammatory indices and hypertension.

**Results:**

Shift work was associated with hypertension [two-shift: risk ratio (RR) 1.75, 95% confidence interval (CI) 1.27–2.05; three-shift: RR 1.75, 95% CI 1.10–2.34; four-shift: RR 1.57, 95% CI 1.10–2.14] and elevated immune-inflammatory index [logarithmic (ln) Systemic Immune-Inflammation Index (SII) 0.18, 95% CI 0.10–0.26, Pan-Immune-Inflammation Value (PIV) 0.15, 95% CI 0.06–0.23, neutrophil-lymphocyte ratio (NLR) 0.17, 95% CI 0.10–0.24, Systemic Inflammation-Response Index (SIRI) 0.14, 95% CI 0.06–0.22, and platelet-lymphocyte ratio (PLR) 0.05, 95% CI 0.01–0.09]. Cumulative night shifts were associated with hypertension (1–220 nights: RR 2.57, 95% CI 1.26–5.26; 220–660 nights: RR 1.78, 95% CI 1.08–2.95; ≥660 nights: RR 2.02, 95% CI 1.22–2.13). Cumulative night shift exposure levels exerted varying effects impacted immune-inflammatory indices. The SII, PIV, NLR, SIRI, and monocyte-lymphocyte ratio (MLR) were independently associated with hypertension risk (RR 1.82, 95% CI 1.47–2.22; RR 1.85, 95% CI 1.53–2.19; RR 2.04, 95% CI 1.60–2.54; RR 2.03, 95% CI 1.64–2.48; RR 1.85, 95% CI 1.27–2.59). The mediating effects of ln(SII), ln(PIV), ln(NLR), ln(SIRI), and composite inflammatory index were 17.52%, 17.51%, 17.78%, 18.64%, and 21.94%, respectively.

**Conclusion:**

Shift work and elevated immune-inflammatory indices were associated with hypertension. Immune-inflammatory indices mediate the association between shift work and hypertension. Therefore, hypertension monitoring should be strengthened among shift workers and immune-inflammatory markers should be considered for early hypertension detection. Future targeted intervention trials are warranted.

Hypertension is a major risk factor for cardiovascular and cerebrovascular diseases (1). By 2023, the global hypertensive population was 1.3 billion, with prevalence projected to reach 44% by 2030 (1−3). Hypertension incidence is increasing worldwide, with a 46.7% adult prevalence in the US (4), 35.5% in India (5), and 245 million affected adults in China (6). This growing burden underscores the necessity of further research into the etiology of hypertension.

In a “24/7” society, shift work accounts for approximately 25% of the US workforce, 20% in Europe, 16% in Australia (7–9), and about 30% in China (10). Previous research on the association between shift work and hypertension has yielded conflicting results. Cohort studies from Canada, China, and Japan have confirmed that shift work was associated with an increased risk of hypertension (11–13). However, other cohort studies found no such association (14, 15). In addition, Yang (16) showed that two-shift, three-shift, and four-shift systems all increased the risk of hypertension onset, while other investigations (17, 18) failed to identify a significant association between these shift patterns and hypertension. Therefore, the associations among shift work, different shift patterns, and hypertension still require further investigation.

Studies have shown that the pathogenesis of hypertension may be mediated by autoimmune, inflammatory, and metabolic abnormalities (19). Leukocytes, their subsets, and platelets are key components of the inflammatory response of the body. Unlike single inflammatory parameters that are susceptible to confounding factors, the neutrophil-to-lymphocyte ratio (NLR) reflects persistent inflammation and immune regulatory pathway activation (20). The platelet-to-lymphocyte ratio (PLR) reflects platelet and lymphocyte count changes in acute inflammation and pro-thrombotic states (21). The monocyte-to-lymphocyte ratio (MLR) targets monocyte-mediated chronic inflammation (22). The Systemic Immune-Inflammation Index (SII), Systemic Inflammation-Response Index (SIRI), and Pan-Immune-Inflammation Value (PIV) have been shown to optimally predict cardiovascular diseases (23), preeclampsia (24), and central nervous system lymphoma (25), respectively. The cross-sectional NHANES study showed a positive correlation among SII, NLR, and the prevalence of hypertension, while PLR was not correlated. SII can be a systemic inflammatory marker of high blood pressure (26, 27). A prospective cohort study reported that SII and SIRI predicted the occurrence of hypertension (28), while additional cohort studies confirmed an association between NLR and hypertension (29, 30). However, a reference range for the association of immune-inflammatory index with hypertension has not been established.

Studies found shift workers had significantly higher IL-10 and white blood cell levels (31), as well as elevated C-reactive protein levels (32). However, studies have also reported that white blood cell counts (33), and total lymphocyte counts (34) were significantly lower among shift workers compared to daytime workers. These results suggest that shift work may be associated with immune-inflammatory responses. Moreover, no studies have identified associations between shift work and composite immune-inflammatory indices, nor examined whether immune-inflammatory indices mediate the association between shift work and hypertension.

This study aimed to elucidate the relationships between shift work, immune-inflammatory indices, and hypertension, as well as characterize the mediating effect of these on immune-inflammatory indices. In this study, we hypothesized that (i): shift work is correlated with hypertension; different shift patterns may exert differential effects on the incidence of hypertension (ii); higher immune-inflammatory index levels are associated with increased incidence of hypertension; and (iii) immune-inflammatory indices maybe a mediator between shift work and hypertension.

## Methods

### Study population

Baseline data for this cohort study were collected between 2013 and 2015. This study was part of the Occupational Health Study of Petroleum Industry Workers (OHSPIW), which investigated the effects of occupational risk factors on health (35). Based on the “Classification Catalog of Petrochemical Occupations in China,” stratified sampling techniques were used to randomly select five petrochemical companies. All employees of each company were divided into four groups, totaling 20 groups. Each group was numbered, and 10 groups were randomly selected using the random number table method. A total of 2100 individuals were selected for the study. They underwent health examinations at the Karamay City Disease Prevention and Control Center in Xinjiang and completed a questionnaire containing basic information. Informed consent was obtained from all participants. The study was approved by the Ethics Committee of Nantong University (2013-L073).

Sample size was calculated according to the following formula:


$$n\text{=}\frac{{\left(Z_{1\text{-}\alpha \text{/}2}\sqrt{2\overset{¯}{\mathrm{pq}}}\text{+}Z_{\beta }\sqrt{p_{0}q_{0}\text{+}p_{1}q_{1}}\right)}^{2}}{{\left(p_{1}\text{-}p_{0}\right)}^{2}}$$

Referring to previous literature, P0=0.128 (18), P1=0.2049 (16), α=0.05, β 0.10. According to the formula, the final sample size required 982 active employees.

To ensure accuracy, we screened the study population using the following inclusion criteria to ensure participants: (i) had worked in their current position for ≥1 year, (ii) were aged 20–60 years, and (iii) had signed an informed consent form. Participants were excluded if they (i) had a history of hypertension, (ii) were taking antihypertensive medications (N=408), (iii) had conditions affecting blood pressure, (iv) were taking medications affecting blood pressure (N=50), iv) had a history of inflammatory disease (N=90), (vi) had no available leukocyte data (N=70), (vii) answered <80% of the questionnaire (N=78), or (viii) had left their jobs and were unable to work during follow-up (N=82), (supplementary material, https://www.sjweh.fi/article/4287, figure S1). The final follow-up of this study was completed in 2023. Questionnaire surveys and occupational health examinations were carried out at the Karamay Disease Prevention and Control Center in Xinjiang (hereafter the Center). The study cohort included 1322 participants, comprising 753 males and 569 females; 732 were shift workers, and 590 were non-shift workers.

### Immune-inflammatory index measurement

At baseline, (following an overnight fast of 8−12 hours) subjects were brought to the Center between 07:00–8:30 hours to have peripheral venous blood drawn. In particular, shift workers underwent the physical examination only if they had taken a full day off following their shift prior. Complete blood count tests were performed at the Center within 2 hours after blood collection, and the same fully automated hematology analyzer was used to measure neutrophil count, lymphocyte count, platelet count, and monocyte count. Then, we calculated the SII = platelet count × neutrophil count/lymphocyte count; the PIV = SII × monocyte count; the SIRI = monocyte count × neutrophil count/lymphocyte count; the NLR = neutrophil count ÷ lymphocyte count; the MLR = monocyte count / lymphocyte count; and the PLR = platelet count / lymphocyte count.

### Diagnostic criteria for hypertension

As above, participants arrived at the Center (07:00–08:30 hours) after an 8–12-hour fast, then rested in a seated position ≥5 minutes (upper arms at heart level), and had their blood pressure measured via a calibrated Omron HEM-7211 upper-arm electronic sphygmomanometer (mmHg). Two readings were taken at 1−2-minute intervals (mean recorded); a third was added if systolic/distolic blood pressure (SBP/DPB) differences were >5 mmHg, with the mean of three used. Hypertension is defined according to the Chinese Guidelines for the Prevention and Treatment of Hypertension (2018 Revised Edition) (36): SBP ≥140 mm Hg and/or DPB ≥90 mm Hg. At follow-up, for participants who diagnosed with hypertension or on antihypertensive treatment, their initial medical records were retrieved and verified according to a unified standard (SBP ≥140 mmHg and/or DBP≥90 mmHg).

### Shift work and cumulative night shifts

This study obtained shift information through questionnaire surveys (37) and simultaneously consulted the company’s schedule to collect the monthly night shift frequency of research subjects. In accordance with the International Labor Organization (ILO) Convention Concerning Night Work (No. 171) (38), shift work involving night shifts was defined as work that lasted for ≥7 consecutive hours and included the period from 24:00–05:00 hours. Participants were divided into two groups: day and shift workers. Day workers followed a fixed schedule from 08:00–20:00 hours. Shift workers adopted variable schedules, including three types of rotations: two-shift rotations (08:00–20:00 and 20:00–08:00, with weekly alternations); three-shift two-rotations (08:00–20:00, 20:00–08:00, and rest periods in sequence); and four-shift, three-rotations [8-hour morning shifts (08:00–16:00), 8-hour afternoon shifts (16:00–24:00), and 8-hour evening shifts (00:00–08:00) in scheduled sequences]. All shift workers had worked ≥3 night shifts monthly for >1 year before the study.

Based on the study by Jankowiak et al (39), cumulative night shifts were calculated using the following formula: number of monthly night shifts × 11 months (deducting approximately 1 month of annual leave) × years of work. Subsequently, the total cumulative number of night shifts was categorized as follows: (i) 0 (control group), (ii) 1–220 (low exposure), (iii) 220–660 (moderate exposure), and (v) ≥660 nights (high exposure).

### Covariates

We collected baseline data using a self-administered questionnaire (37), which included (i) age: 20−30, 30−40, and 40−60 years; (ii) Chinese standard body mass index (BMI): <18.5 (underweight), 18.5−23.9 (normal weight), 24−28.0 (overweight), and ≥28 (obese) kg/m^2^; (iii) ethnicity: Han, Uyghur, and other minority groups; (iv) marital status: unmarried, married, and other (including divorced, widowed, or remarried); (v) educational attainment: high school or below, college or above, and other; (vi) monthly income: <3000, 3000–5000, and ≥5000 yuan; (vii) smoking: ≥1 (often), <1 (occasional) cigarettes/day, quit smoking, and non-smoker; (viii) alcohol consumption: ≥8 (often), <8 (occasional), and 0 (non-drinker) grams/day; (ix) physical exercise is categorized as 'yes' or 'no'. In line with the Chinese Diabetes Diagnostic Guidelines 2022, participants were classified as diabetic and non-diabetic (40). Family history of hypertension was categorized as 'yes' or 'no'. Family history of diabetes is categorized as 'yes' or 'no';(x) Length of employment is categorized as <10, 10−20, 20−30, and ≥30 years.

### Statistical analysis

Questionnaire data were entered into a database using EpiData 3.0 software with dual data entry. Data were organized and analyzed using SPSS (version 26.0), R (version 4.3.3), and STATA/MP (version 17.0) statistical software. For descriptive analysis, continuous variables were presented as the mean [standard deviation (SD)] or median [interquartile range (IQR)], and categorical variables as frequency (percentage), with chi-square tests and t-tests applied for respective analyses. Considering the skewed distribution of the immune-inflammatory index, the six systemic inflammation indices were ln-transformed and grouped using quartiles to investigate their association with hypertension incidences. Uni- and multivariate logistic regression models were used: unadjusted (model 1); adjusted for sex, age, ethnicity, BMI, education level, marital status, and monthly income (model 2); and additionally adjusted for smoking, drinking, physical exercise, diabetes, family history of diabetes, family history of hypertension, and length of employment (model 3). To further illustrate the associations among immune-inflammatory indices and hypertension, we performed smooth curve-fitting analysis based on model 3 and used a piecewise linear regression model to explore the threshold effect between them. The immune-inflammatory index was segmented based on the inflection point, and we used the Karlson-Holm-Breen method to evaluate the mediation effect of immune-inflammatory indices on the association between shift work and hypertension. All statistical tests in this study were two-sided, with a significance level of α=0.05.

## Results

This study included 1322 participants aged 20–60 years, of whom 56.96% were men and 43.04% were women. Of thes participants, 732 (55.37%) were shift workers. During the study period, 217 cases of hypertension occurred, with an incidence of 16.41%. Results showed a statistically significant difference in shift work distribution between different smoking and exercise groups (P<0.05). However, no significant difference was observed for sex, age, ethnicity, BMI, educational level, monthly income, drinking, and family history of diabetes, hypertension, length of employment, or other factors (P>0.05) (table 1).

**Table 1:** Distribution of shift work and hypertension among different demographic characteristics

Variable		Shift work N (%)		P-value
		No (N=590)	Yes (N=722)	
Sex				0.498
	Male	330 (43.82)	423 (56.18)	
	Female	260 (45.69)	309 (54.31)	
Age (year)				0.334
	20–30	43 (38.74)	68 (61.26)	
	30–40	255 (46.39)	260 (53.61)	
	40–60	322 (44.35)	404 (55.65)	
Ethnicity				0.267
	Han	454 (45.00)	556 (55.00)	
	Uygur	67 (48.55)	71 (51.45)	
	Other	69 (39.66)	105 (60.34)	
Body mass index (kg/m^2^)				0.666
	18.5–23.9	298 (45.85)	352 (54.15)	
	≤18.5	21 (50.00)	22 (50.00)	
	23.9–28	206 (42.83)	275 (57.17)	
	≥28	65 (43.62)	84 (56.38)	
Education level				0.209
	High school or below	195 (48.03)	211 (51.97)	
	College or above	256 (42.38)	348 (57.62)	
	Unknown	139 (44.60)	173 (55.40)	
Marital status				0.134
	Not married	152 (47.80)	166 (52.20)	
	Married	330 (45.08)	402 (54.92)	
	Other	108 (39.71)	164 (60.29)	
Monthly income (yuan)				0.526
	<3000	149 (44.21)	188 (55.79)	
	3000–5000	355 (45.69)	422 (54.31)	
	≥5000	86 (41.35)	122 (58.65)	
Smoking				0.001
	No	407 (42.71)	546 (57.29)	
	Often	123 (44.24)	155 (55.76)	
	Occasional	49 (67.12)	24 (32.98)	
	Quit smoking	7 (53.85)	6 (46.15)	
Drinking				0.120
	No	379 (42.63)	510 (57.37)	
	Often	28 (47.46)	31 (52.54)	
	Occasional	182 (48.79)	191 (51.21)	
Physical exercise				0.007
	No	293 (48.67)	309 (51.33)	
	Yes	297 (41.25)	423 (58.75)	
Diabetes				0.259
	No	353 (43.53)	458 (56.47)	
	Yes	33 (54.10)	28 (45.90)	
	Unknown	204 (45.33)	246 (54.67)	
Family history of hypertension				0.294
	No	583 (44.81)	718 (55.19)	
	Yes	7 (33.33)	14 (66.67)	
Family history of diabetes				0.279
	No	585 (44.79)	721 (55.21)	
	Yes	5 (31.25)	11 (68.75)	
Length of Employment (years)				0.60
	<10	142 (40.34)	210 (59.66)	
	10–20	139 (42.39)	189 (57.61)	
	20–30	213 (46.92)	241 (53.08)	
	≥30	96 (51.06)	92 (48.94)	

Table 2 presents analysis of the association between shift work, cumulative night shifts, and hypertension. In model 1, the incidence risk for shift workers was 1.67 times higher than that of the fixed day shift group [95% confidence interval (CI) 1.21−2.00]. The risks for two-, three-, and four-shift workers were 1.79 times (95% CI 1.33−2.36), 1.66 times (95% CI 1.07−2.47), and 1.54 times (95% CI 1.11−2.08), respectively. Compared with the control group, the relative risks (RR) for workers with 1–220, 220–660, and ≥660 nights were 1.87 (95% CI 0.96−3.63), 1.51 (95% CI 0.95−2.40), and 2.09 (95% CI 1.55−2.81), respectively. In model 3, the RR for shift workers was 1.67 times that of the fixed day shift group (95% CI 1.28−1.91). Two-shift workers had a RR of 1.75 (95% CI 1.27−2.05), three-shift workers (RR 1.75, 95% CI 1.10−2.34), and four-shift workers (RR 1.57, 95% CI 1.10−2.14) had higher incidence risks than the fixed day shift group. The analysis of cumulative night shifts in this model identified RR of 2.57 (95% CI 1.26−5.26), 1.78 (95% CI 1.08−2.95), 2.02 (95% CI 1.22−2.13) for 1–220, 220–660, and ≥660 nights, respectively.

**Table 2:** Association between shift work, cumulative night shifts and hypertension incidence. [RR=risk ratio; CI=confidence interval

Variable		Incidence of hypertension	Model 1 ^a^			Model 2 ^b^			Model 3 ^c^	
			RR (95% CI)	P-value		RR (95% CI)	P-value		RR (95% CI)	P-value
Fixed day shift		72/590	1.00			1.00			1.00	
Shift work		145/732	1.67 (1.21–2.00)	<0.001		1.70 (1.30–2.19)	<0.001		1.67 (1.28–1.91)	<0.001
Shift work type										
	Fixed day	72/590	1.00			1.00			1.00	
	2-shift rotation	68/253	1.79 (1.33–2.36)	<0.001		1.80 (1.32–2.40)	<0.001		1.75 (1.27–2.05)	0.001
	3-shift rotation	23/113	1.66 (1.07–2.47)	0.025		1.74 (1.10–2.63)	0.021		1.75 (1.10–2.34)	0.021
	4-shift rotation	54/298	1.54 (1.11–2.08)	0.010		1.58 (1.12–2.16)	0.009		1.57 (1.10–2.14)	0.012
Cumulative night shifts										
	0	72/590	1.00			1.00			1.00	
	1–220	10/50	1.87 (0.96–3.63)	0.064		1.97 (0.99–3.91)	0.052		2.57 (1.26–5.26)	0.009
	220–660	24/140	1.51 (0.95–2.40)	0.080		1.66 (1.04–2.66)	0.034		1.78 (1.08–2.95)	0.025
	≥660	111/542	2.09 (1.55–2.81)	<0.001		2.09 (1.55–2.83)	0.001		2.02 (1.22–2.13)	<0.001

^a^ Unadjusted. ^b^ Adjusted for sex, age, ethnicity, BMI, education level, marital status, and monthly income. ^c^ Adjusted for gender, age, ethnicity, BMI, education level, marital status, monthly income, smoking, drinking, physical exercise, diabetes, family history of diabetes, family history of hypertension, and length of employment.

Table 3 shows the effects of shift work on the immunological inflammation index (ln transformation). Model 1 showed that shift workers had significantly higher ln(SII) (β 0.18, 95% CI 0.10−0.25), ln(PIV) (β 0.14, 95% CI 0.06−0.23), ln(NLR) (β 0.16, 95% CI 0.09−0.24), ln(SIRI) (β 0.14, 95% CI 0.06−0.21), and ln(PLR) (β 0.05, 95% CI 0.01−0.08). After adjusting for all confounding factors in model 3, shift workers showed average increases of 0.18 units in ln(SII) (95% CI 0.10−0.22), 0.15 units in ln(PIV) (95% CI 0.06−0.23), 0.17 units in ln(NLR) (95% CI 0.10−0.24), 0.14 units in ln(SIRI) (95% CI 0.06−0.22), and 0.05 units in ln(PLR) (95% CI 0.01−0.09). However, the difference in ln(MLR) between the shift work group and the fixed day shift group was not statistically significant. After adjusting for all confounding factors, the high-exposure group (> 660 nights) demonstrated significantly higher levels of ln(SII) (β 0.20, 95% CI 0.11−0.28), ln(PIV) (β 0.16, 95% CI 0.11−0.28), ln(NLR) (β 0.19, 95% CI 0.11−0.26), and ln(SIRI) (β 0.16, 95% CI 0.07−0.24) than the control group (supplementary tables S1−S6).

**Table 3:** Association between shift work and immune inflammation indices. [CI=confidence interval; SE=standard error; ln=logarithmic; SII=Systemic Immune-Inflammation Index; PIV=Pan Immune-Violation Index; NLR=neutrophil-lymphocyte ratio; SIRI=Systemic Inflammatory-Response Index; PLR=platelet-lymphocyte ratio; MLR=monocyte-lymphocyte ratio.]

Model		Variable	β (95% CI)	SE	t	P-value
ln (SII)						
	Model 1 ^a^	Fixed day shift	0.00			
		Shift work	0.18 (0.10–0.25)	0.04	4.50	<0.001
	Model 2 ^b^	Shift work	0.18 (0.10–0.26)	0.04	4.61	<0.001
	Model 3 ^c^	Shift work	0.18 (0.10–0.26)	0.04	4.61	<0.001
ln (PIV)						
	Model 1 ^a^	Fixed day shift	0.00			
		Shift work	0.14 (0.06–0.23)	0.04	3.35	0.001
	Model 2 ^b^	Shift work	0.15 (0.06–0.23)	0.04	3.41	0.001
	Model 3 ^c^	Shift work	0.15 (0.06–0.23)	0.04	3.41	0.009
ln (NLR)						
	Model 1 ^a^	Fixed day shift	0.00			
		Shift work	0.16 (0.09–0.24)	0.04	4.60	<0.001
	Model 2 ^b^	Shift work	0.17 (0.10–0.24)	0.04	4.80	<0.001
	Model 3 ^c^	Shift work	0.17 (0.10–0.24)	0.04	4.79	<0.001
ln (SIRI)						
	Model 1 ^a^	Fixed day shift	0.00			
		Shift work	0.14 (0.06–0.21)	0.04	3.34	0.001
	Model 2 ^b^	Shift work	0.14 (0.06–0.22)	0.04	3.48	0.001
	Model 3 ^c^	Shift work	0.14 (0.06–0.215)	0.04	2.46	0.001
ln (MLR)						
	Model 1 ^a^	Fixed day shift	0.00			
		Shift work	0.01 (-0.03–0.05)	0.02	0.38	0.704
	Model 2 ^b^	Shift work	0.01 (-0.03–0.04)	0.02	0.33	0.741
	Model 3 ^c^	Shift work	0.01 (-0.01–0.01)	0.02	0.32	0.748
ln (PLR)						
	Model 1 ^a^	Fixed day shift	0.00			
		Shift work	0.05 (0.01–0.09)	0.02	2.63	0.009
	Model 2 ^b^	Shift work	0.05 (0.19–0.88)	0.02	2.62	0.009
	Model 3 ^c^	Shift work	0.05 (0.01–0.09)	0.01	2.67	0.008

^a^ Model 1: Unadjusted. ^b^ Model 2: Adjusted for sex, age, ethnicity, BMI, education level, marital status, and monthly income. ^c^ Model 3: Adjusted for gender, age, ethnicity, BMI, education level, marital status, monthly income, smoking, drinking, physical exercise, diabetes, family history of diabetes, family history of hypertension, and length of employment.

Table 4 shows that the ln-transformed immune-inflammatory indices [ln(SII), ln(PIV), ln(NLR), ln(SIRI), and ln(MLR)] were all positively associated with the risk of hypertension. This association remained significant in models 1, 2, and 3. Model 3 revealed that individuals with higher levels of these five indicators had a significantly increased risk of hypertension onset: an 82% increase in ln(SII) (RR 1.82, 95% CI 1.47−2.22), an 85% increase in ln(PIV) (RR 1.85, 95% CI 1.53−2.19), an 104% increase in ln(NLR) (RR 2.04, 95% CI 1.60−2.54), with ln(SIRI) increased by 103% (RR 2.03, 95% CI 1.64−2.48), and ln(MLR) increased by 85% (RR 1.85, 95% CI 1.27−2.59). After grouping by quartiles in model 3, with adjustment for all confounding factors, the dose–response relationship remained significant. The risks for ln(SII) in Q2, Q3, and Q4 were 50%, 52%, and 122% higher than in Q1, respectively. The risks for ln(PIV) in Q3 and Q4 were 103% and 130% higher, respectively. The risks for ln(NLR) in Q2, Q3, and Q4 were 89%, 102%, and 148% higher, respectively. The risks for ln(SIRI) in Q2, Q3, and Q4 were 56%, 108%, and 175% higher, respectively. The risks for ln(MLR) in Q3 and Q4 were 59% and 74% higher, respectively. Additionally, for all models the quantiles (Q2, Q3, and Q4) for ln(PLR) were not significantly associated with hypertension incidence.

**Table 4:** Association between immune inflammation index and hypertension incidence. [RR=risk ratio; CI=confidence interval; ln=logarithmic; SII=Systemic Immune-Inflammation Index; PIV=Pan Immune-Violation Index; NLR=neutrophil-lymphocyte ratio; SIRI=Systemic Inflammatory-Response Index; PLR=platelet-lymphocyte ratio; MLR=monocyte-lymphocyte ratio.]

Variable		Incidence of hypertension		Model 1 ^a^			Model 2 ^b^			Model 3 ^c^	
		N (%)		RR (95% CI)	P-value		RR (95% CI)	P-value		RR (95% CI)	P-value
ln (SII)		217 (16.41)		1.74 (1.42–2.11)	<0.001		1.82 (1.47–2.22)	<0.001		1.82 (1.47–2.22)	<0.001
ln (SII) categories											
	≤5.59	37 (11.17)		1.00			1.00			1.00	
	5.59–6.00	54 (16.36)		1.44 (0.99–2.03)	0.054		1.53 (1.04–2.16)	0.03		1.50 (1.01–2.93)	0.04
	6.00–6.34	50 (15.11)		1.33 (0.91–1.90)	0.136		1.52 (1.03–2.16)	0.039		1.52 (1.03–2.13)	0.039
	>6.34	76 (23.03)		1.99 (1.44–2.65)	<0.001		2.20 (1.58–2.16)	<0.001		2.22 (1.55–3.03)	<0.001
ln (PIV)		217 (16.41)		1.66 (1.38–3.03)	<0.001		1.83 (1.50–1.98)	<0.001		1.85 (1.53–2.19)	<0.001
ln (PIV) categories											
	≤4.57	36 (10.90)		1.00			1.00			1.00	
	4.57–5.02	42 (12.65)		1.15 (0.76–1.72)	0.487		1.18 (0.76–2.71)	0.447		1.18 (0.76–3.08)	0.429
	5.02–5.43	66 (20.06)		1.82 (1.27–1.71)	0.001		2.01 (1.40–1.76)	<0.001		2.03 (1.41–1.78)	<0.001
	>5.43	73 (22.05)		2.00 (1.41–2.51)	<0.001		2.27 (1.60–2.78)	<0.001		2.30 (1.62–2.83)	<0.001
ln (NLR)		217 (16.41)		1.95 (1.54–3.14)	<0.001		2.04 (1.60–2.43)	<0.001		2.04 (1.60–2.54)	<0.001
ln (NLR) categories											
	≤0.21	31 (9.39)		1.00			1.00			1.00	
	0.21–0.54	56 (16.77)		1.74 (1.18–2.56)	0.005		1.88 (1.27–3.04)	0.002		1.89 (1.27–3.35)	0.002
	0.54–0.85	59 (17.72)		1.84 (1.26–2.47)	0.002		1.98 (1.34–2.68)	0.001		2.02 (1.37–2.70)	0.001
	>0.85	71 (21.85)		2.24 (1.57–2.58)	<0.001		2.47 (1.73–2.80)	<0.001		2.48 (1.73–3.39)	<0.001
ln (SIRI)		217 (16.41)		1.78 (1.46–3.39)	<0.001		1.97 (1.60–2.40)	<0.001		2.03 (1.64–2.48)	<0.001
ln (SIRI) categories											
	≤-0.84	31 (9.31)		1.00			1.00			1.00	
	-0.84– -0.45	46 (14.94)		1.57 (1.04–2.28)	0.03		1.51 (0.98–2.22)	0.059		1.56 (1.02–2.30)	0.042
	-0.45– -0.04	61 (17.23)		1.80 (1.24–2.53)	0.003		2.00 (1.36–2.80)	0.001		2.08 (1.41–2.92)	<0.001
	>-0.04	79 (24.16)		2.47 (1.77–3.30)	<0.001		2.66 (1.89–3.56)	<0.001		2.75 (1.95–3.68)	<0.001
ln (MLR)		217 (16.41)		1.42 (0.99–1.98)	0.051		1.81 (1.24–2.53)	0.002		1.85 (1.27–2.59)	0.002
ln (MLR) categories											
	≤-1.97	43 (12.22)		1.00			1.00			1.00	
	-1.97– -1.71	48 (18.46)		1.51 (1.03–2.14)	0.033		1.47 (0.99–2.11)	0.058		1.46 (0.98–2.11)	0.063
	-1.71– -1.47	77 (18.25)		1.49 (1.06–2.05)	0.022		1.60 (1.12–2.21)	0.01		1.59 (1.12–2.21)	0.011
	>-1.47	49 (17.01)		1.39 (0.96–1.98)	0.086		1.70 (1.16–2.42)	0.007		1.74 (1.18–2.47)	0.006
ln (PLR)		217 (16.41)		1.16 (0.79–1.67)	0.428		1.35 (0.91–1.95)	0.136		1.34 (0.90–1.94)	0.145
ln (PLR) categories											
	≤4.47	58 (17.52)		1.00			1.00			1.00	
	4.47–4.70	60 (18.18)		1.05 (0.73–1.46)	0.825		1.12 (0.77–1.59)	0.531		1.09 (0.75–1.55)	0.642
	4.80–4.91	42 (12.80)		0.71 (0.47–1.04)	0.084		0.84 (0.55–1.24)	0.387		0.83 (0.54–1.23)	0.36
	>4.91	57 (17.12)		0.98 (0.68–1.39)	0.932		1.08 (0.74–1.55)	0.683		1.06 (0.72–1.52)	0.764

^a^ Model 1: Unadjusted. ^b^ Model 2: Adjusted for sex, age, ethnicity, BMI, education level, marital status, and monthly income. ^c^ Model 3: Adjusted for gender, age, ethnicity, BMI, education level, marital status, monthly income, smoking, drinking, physical exercise, diabetes, family history of diabetes, family history of hypertension, and length of employment.

The results of the smooth curve fitting analysis are available in supplementary figure S2 (a, b, c, d, f), which shows that ln(SII), ln(PIV), ln(NLR), ln(SIRI), and ln(PLR) exhibited a nonlinear association with hypertension incidence, whereas supplementary figure S2e shows that ln(MLR) exhibited a linear association with hypertension incidence.

The results of threshold effect analysis revealed the following inflection point values for each indicator: ln(SII)=4.90, ln(PIV)=4.01, ln(NLR)=-0.40, ln(SIRI)=-1.24, ln(PLR)=5.20, and ln(MLR)=-1.83 (supplementary table S7).

Next, severe multicollinearity was detected among the four inflammatory indices ln(SII), ln(PIV), ln(NLR), and ln(SIRI). To address this issue, we performed factor analysis to construct a composite inflammatory index (supplementary tables S8–10). The results of mediation analysis showed that all four indices had an indirect influence on the positive correlation between shift work and hypertension incidence (table 5). Among these, the mediating effect of ln(SIRI) was 18.64% (P<0.001), ln(SII) 17.52% (P<0.001), ln(PIV) 17.51% (P=0.001), ln(NLR) 17.78% (P<0.001), with the composite inflammatory index 21.94% (P<0.001).

**Table 5:** Mediating effect of immune inflammation index (ln transformation) between shift work (exposure) and hypertension (outcome). Adjusted for gender, age, ethnicity, body mass index, education level, marital status, monthly income, smoking, drinking, physical exercise, diabetes, family history of diabetes, family history of hypertension, and length of employment. [SE=standard error; ln=logarithmic; SII=Systemic Immune-Inflammation Index; PIV=Pan Immune-Violation Index; NLR=neutrophil-lymphocyte ratio; SIRI=Systemic Inflammatory-Response Index; PLR=platelet-lymphocyte ratio; MLR=monocyte-lymphocyte ratio.]

Mediator	Direct effect	SE	P-value	Indirect effect	SE	P-value	Total effect	SE	P-value	Proportion mediation (%)
ln(SII)	0.52	0.17	0.002	0.11	0.05	0.043	0.63	0.17	<0.001	17.52
ln(PIV)	0.52	0.17	0.002	0.11	0.05	0.030	0.64	0.17	<0.001	17.51
ln(NLR)	0.51	0.17	0.002	0.11	0.05	0.042	0.63	0.17	<0.001	17.78
ln(SIRI)	0.51	0.17	0.002	0.12	0.06	0.035	0.63	0.17	<0.001	18.64
Composite inflammatory index	0.48	0.17	0.005	0.14	0.04	0.001	0.62	0.17	<0.001	21.94

## Discussion

This study showed that shift work was associated with an increased risk of hypertension and elevated immune-inflammatory indices. Furthermore, high levels of immune-inflammatory indices were associated with hypertension. Ln(SII), ln(PIV), ln(NLR), ln(SIRI), and ln(PLR) exhibited positive nonlinear correlations with hypertension incidences. The ln(MLR) demonstrated a positive linear correlation with hypertension incidences. Further, cumulative night shifts increased hypertension risk and elevated immune-inflammatory indices. Ln(SII), ln(PIV), ln(NLR), and ln(SIRI) played a partial mediating role in the association between shift work and hypertension.

Shift work was significantly associated with an increased risk of hypertension, consistent with other cohort study findings (11, 13). Consistent with the findings of Yang et al (16), this study found that two-, three-, and four-shift systems were linked to elevated hypertension risk. However, the studies conducted by Hublin et al (14) and Biggi et al (15) did not find an association between shift work and the risk of hypertension. These discrepancies may stem from the research questionnaires used in the previous two studies: participants were only asked whether they worked night, day, or rotating shifts, without further specifying the exact types of rotating shifts (such as fixed night shifts, two-shift rotations, three-shift two-day rotations, etc.). Shift work can disrupt circadian rhythms (disrupting the biological clock regulated by CLOCK and BMAL1), leading to abnormal blood pressure (failure of nighttime blood pressure to decrease) and hormonal release disorders (41). This study also found that the cumulative number of night shifts was associated with an increased risk of hypertension incidence. Zhao et al (42) reported a dose–response relationship between cumulative monthly shift work exposure and the risk of hypertension. Meanwhile, Lieu et al (43) found that among Black women, a dose–response relationship existed between the cumulative number of years of rotating night shifts (stratified as 0, 1–4, 5–9, 10–14, and ≥15 years) and hypertension risk. However, the study of Zhao et al was cross-sectional with limited causal inference strength, while the Lieu et al included only women and the association was statistically significant solely in the Black female subgroup. Our study found no dose–response relationship, possibly due to the shift adaptation of workers and inconsistent classification of cumulative shift exposures. Thus, early interventions for shift workers and optimized company work scheduling are recommended to reduce hypertension risk. Additionally, the association between long-term cumulative shift work exposure and hypertension warrants further investigation.

The present study found that the levels of immune-inflammatory indices were higher among shift workers than fixed day-shift workers. Although there is no previous study on the direct correlation between shift work and iimmune-inflammatory indices, it has been widely confirmed that shift work disrupts the physiological rhythms of the body. For example, the circadian rhythm disorder caused by shift work can affect the secretion rhythm of hormones such as cortisol (44) and melatonin (45), which are not only involved in metabolic regulation but also closely related to high-sensitivity C-reactive protein-mediated systemic low-grade inflammation (46). Additionally, animal studies indicate that shift work significantly disrupts lipopolysaccharide-induced acute inflammatory responses in rats (47). Therefore, in occupational health management, companies should prioritize testing the iimmune-inflammatory indices of shift workers and conduct more comprehensive assessments of health risks associated with shift work.

We also found that elevated baseline iimmune-inflammatory markers were associated with an increased risk of hypertension. Participants with higher levels of SII, PIV, NLR, SIRI, and MLR were more likely to develop hypertension. This was consistent with the conclusions of previous cohort studies (28–30). Furthermore, this study preliminarily identified the critical reference range of immune-inflammatory indices associated with hypertension risk, providing practical reference for subsequent clinical screening and risk stratification in relevant populations.

We also found that immune-inflammatory indices mediated the association between shift work and hypertension. Sleep deprivation and the stress associated with shift work significantly elevated levels of pro-inflammatory cytokines (TNF-α, IL-1β, IL-6) (31) and monocytes, lymphocytes, and neutrophils in the systemic circulation (48), potentially leading to vascular wall degeneration and sclerosis. Shift work can disrupt the body’s circadian rhythms (11), leading to misalignment in the secretion patterns of chemokines (eg, MCP-1) and growth factors (eg, GM-CSF) (49), further exacerbating inflammatory imbalance. Therefore, immune-inflammatory indices may serve as an early objective biomarker for hypertension among shift workers. As routine blood tests are mandatory in occupational annual health check-ups per the Law of the People’s Republic of China on the Prevention and Control of Occupational Diseases, this index may be an economical, accessible, and low-cost indicator and is thus recommended for early hypertension screening and risk warning among shift workers.

This was the first study to explore the role of the iimmune-inflammatory composite index in association between shift work and hypertension, and preliminarily established the threshold between the baseline immune -inflammation index and the incidence of hypertension. However, some limitations are unavoidable. First, this study did not account for environmental factors (dietary patterns, workplace noise, and high temperatures), sleep quality, work stress, working hours, which may have introduced residual confounding bias and affected the interpretation of the associations between inflammatory indices and hypertension incidence. Future studies are recommended to quantitatively assess these uncontrolled confounders with standardized tools. Furthermore, combined prospective repeated-measure designs with sensitivity analysis will reduce bias and improve result robustness. Second, immune-inflammatory indices were only measured at baseline without tracking their temporal dynamics, which limits causal inferences. Finally, the study population was restricted to petroleum industry workers, which limited the generalizability of the findings. Future research should expand its scope to include shift workers from other industries.

### Concluding remarks

This study found that shift work, immune-inflammatory indices, and cumulative night shifts were all risk factors for hypertension. Furthermore, immune-inflammatory indices partially mediated the association between shift work and hypertension. Therefore, early intervention in shift work schedules is necessary to reduce the risk of hypertension. Furthermore, immune-inflammatory indices may be early screening indicators for hypertension. However, future intervention studies are therefore needed to further validate these findings.

## Supplementary material

Supplementary material

## Data Availability

The datasets used and analyzed during the current study are available from the corresponding author on reasonable request.
