# Supplementary material for: The mediating effect of immune-inflammatory indices in shift work and hypertension: a cohort study in China
Source: Scand J Work Environ Health. 2026 Apr 30;52(3):322–32. doi: 10.5271/sjweh.4287 (PMC13185747; doi:10.5271/sjweh.4287)
Supplement: Supplementary material [file SJWEH-52-322-S001.pdf]

The mediating effect of immune-inflammatory indices in shift work and hypertension: a cohort study in China[1]

by Haixia Lu, Yuxin Jin, Changxue Huang, Lan Lin, Xiangran Zhang, Wanyu Wang, Yulong Lian[2]

1. Supplementary material
2. Correspondence to: Yulong Lian, PhD, Division of Epidemiology and Medical Statistics, School of Public Health, Nantong University, Se Yuan Road, No 9, Nantong, Jiangsu, 226019, China. [E-mail: lianyulong444@163.com]. ORCID: https://orcid.org/0000-0002-6464-6690.

Figure S1

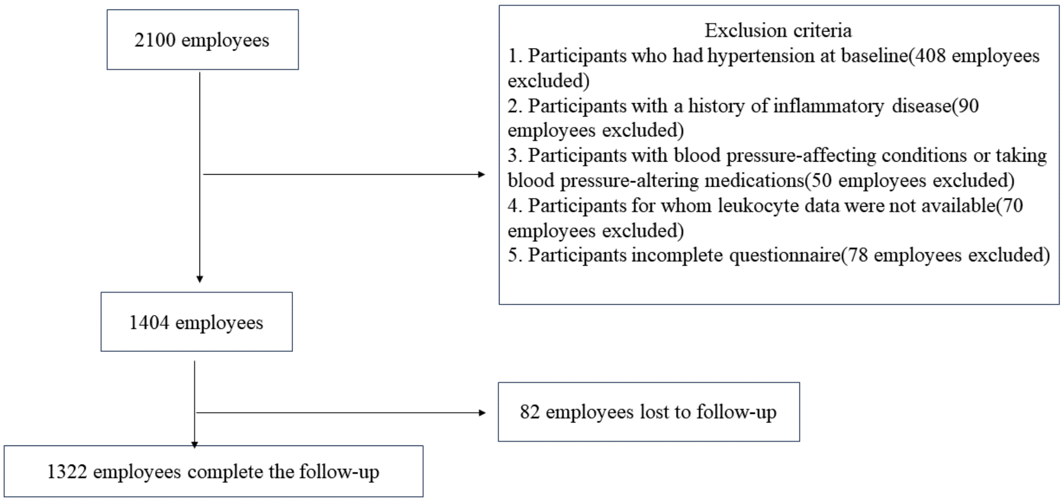

Flow chart of study population

S1 Cumulative night shifts and ln(SII) correlation analysis

Model	Variable	β(95%CI)	SE	t	P
ln (SII)					
Model 1	0 night	0.00			
	1–220 nights	0.15(-0.5-0.35)	0.10	1.44	0.150
	220–660 nights	0.11(-0.02-0.24)	0.07	1.73	0.085
	≥660 nights	0.19(0.11-0.28)	0.04	4.62	<0.001
Model 2	0 night	0.00			
	1–220 nights	0.18(-0.30-0.38)	0.11	1.68	0.093
	220–660 nights	0.12(-0.01-0.25)	0.07	1.75	0.081
	≥660 nights	0.18(0.10-0.27)	0.04	4.38	<0.001
Model 3	0 night	0.00			
	1–220 nights	0.16(-0.5-0.38)	0.11	1.51	0.131
	220–660 nights	0.12(-0.02-0.26)	0.07	1.68	0.093
	≥660 nights	0.20(0.11-0.28)	0.04	4.62	<0.001

Model 1: Unadjusted

Model 2: Adjusted for sex, age, ethnicity, BMI, education level, marital status, and monthly income.

Model 3: Adjusted for gender, age, ethnicity, BMI, education level, marital status, monthly income, smoking, drinking, physical exercise, diabetes, family history of diabetes, family history of hypertension, and length of employment.

S2 Cumulative night shifts and ln(PIV) correlation analysis

Model	Variable	β(95%CI)	SE	t	P
ln (PIV)					
Model 1	0 night	0.00			
	1–220 nights	0.06(-0.16-0.29)	0.13	0.53	0.596
	220–660 nights	0.15(0.001-0.29)	0.07	1.98	0.048
	≥660 nights	0.15(0.06-0.24)	0.05	3.28	0.001
Model 2	0 night	0.00			
	1–220 nights	0.11(-0.3-0.38)	0.12	0.92	0.360
	220–660 nights	0.13(-0.01-0.25)	0.07	1.82	0.069
	≥660 nights	0.14(0.10-0.27)	0.05	3.03	0.002
Model 3	0 night	0.00			
	1–220 nights	0.05(-0.5-0.38)	0.12	0.40	0.690
	220–660 nights	0.11(-0.02-0.26)	0.08	1.39	0.165
	≥660 nights	0.16(0.11-0.28)	0.05	3.52	<0.001

Model 1: Unadjusted

Model 2: Adjusted for sex, age, ethnicity, BMI, education level, marital status, and monthly income.

Model 3: Adjusted for gender, age, ethnicity, BMI, education level, marital status, monthly income, smoking, drinking, physical exercise, diabetes, family history of diabetes, family history of hypertension, and length of employment.

S3 Cumulative night shifts and ln(NLR) correlation analysis

Model	Variable	β(95%CI)	SE	t	P
ln (NLR)					
Model 1	0 night	0.00			
	1–220 nights	0.16(-0.03-0.34)	0.10	1.63	0.102
	221–660 nights	0.10(-0.02-0.22)	0.06	1.66	0.097
	>660 nights	0.18(0.11-0.26)	0.04	4.72	<0.001
Model 2	0 night	0.00			
	1–220 nights	0.18(-0.01-0.36)	0.10	1.82	0.070
	220–660 nights	0.11(-0.01-0.25)	0.06	1.76	0.078
	≥660 nights	0.18(0.10-0.25)	0.04	4.54	<0.001
Model 3	0 night	0.00			
	1–220 nights	0.16(-0.04-0.35)	0.10	1.57	0.116
	220–660 nights	0.11(-0.02-0.23)	0.06	1.66	0.097
	≥660 nights	0.19(0.11-0.26)	0.04	4.83	<0.001

Model 1: Unadjusted

Model 2: Adjusted for sex, age, ethnicity, BMI, education level, marital status, and monthly income.

Model 3: Adjusted for gender, age, ethnicity, BMI, education level, marital status, monthly income, smoking, drinking, physical exercise, diabetes, family history of diabetes, family history of hypertension, and length of employment.

S4 Cumulative night shifts and ln(SIRI) correlation analysis

Model	Variable	β(95%CI)	SE	t	P
ln (SIRI)					
Model 1	0 night	0.00			
	1–220 nights	0.07(-0.14-0.28)	0.11	0.65	0.517
	220–660 nights	0.13(-0.004-0.26)	0.06	1.90	0.057
	≥660 nights	0.14(0.06-0.23)	0.04	3.27	0.001
Model 2	0 night	0.00			
	1–220 nights	0.11(-0.01-0.36)	0.11	1.00	0.319
	220–660 nights	0.12(-0.01-0.25)	0.07	1.80	0.072
	≥660 nights	0.13(0.10-0.25)	0.04	3.07	0.002
Model 3	0 night	0.00			
	1–220 nights	0.04(-0.18-0.26)	0.11	0.38	0.700
	220–660 nights	0.09(-0.05-0.24)	0.07	1.32	0.188
	≥660 nights	0.16(0.07-0.24)	0.04	3.61	<0.001

Model 1: Unadjusted

Model 2: Adjusted for sex, age, ethnicity, BMI, education level, marital status, and monthly income.

Model 3: Adjusted for gender, age, ethnicity, BMI, education level, marital status, monthly income, smoking, drinking, physical exercise, diabetes, family history of diabetes, family history of hypertension, and length of employment.

S5 Cumulative night shifts and ln(MLR) correlation analysis

Model	Variable	β(95%CI)	SE	t	P
ln(MLR)					
Model 1	0 night	0.00			
	1–220 nights	-0.02(-0.12-0.09)	0.05	-0.29	0.773
	220–660 nights	0.04(-0.02-0.11)	0.03	1.25	0.212
	≥660 nights	0.001(-0.04-0.04)	0.02	0.04	0.972
Model 2	0 night	0.00			
	1–220 nights	-0.02(-0.12-0.09)	0.05	-0.29	0.773
	220–660 nights	0.04(-0.02-0.11)	0.03	1.25	0.212
	≥660 nights	0.003(-0.04-0.04)	0.02	0.04	0.972
Model 3	0 night	0.00			
	1–220 nights	-0.05(-0.18-0.26)	0.06	-0.88	0.378
	220–660 nights	0.02(-0.05-0.24)	0.04	0.52	0.604
	≥660 nights	0.01(0.07-0.24)	0.02	0.34	0.733

Model 1: Unadjusted

Model 2: Adjusted for sex, age, ethnicity, BMI, education level, marital status, and monthly income.

Model 3: Adjusted for gender, age, ethnicity, BMI, education level, marital status, monthly income, smoking, drinking, physical exercise, diabetes, family history of diabetes, family history of hypertension, and length of employment.

S6 Cumulative night shifts and ln(PLR) correlation analysis

Model	Variable	β(95%CI)	SE	t	P
ln(PLR)					
Model 1	0 night	0.00			
	1–220 nights	0.06(-0.03-0.16)	0.05	1.31	0.191
	220–660 nights	0.02(-0.04-0.09)	0.03	0.79	0.431
	≥660 nights	0.05(0.01-0.09)	0.02	2.68	0.007
Model 2	0 night	0.00			
	1–220 nights	0.05(-0.05-0.15)	0.05	1.04	0.300
	220–660 nights	0.04(-0.03-0.10)	0.03	1.12	0.263
	≥660 nights	0.06(0.02-0.10)	0.02	2.79	0.005
Model 3	0 night	0.00			
	1–220 nights	0.07(-0.03-0.18)	0.05	1.43	0.152
	220–660 nights	0.04(-0.02-0.11)	0.03	1.27	0.204
	≥660 nights	0.05(0.01-0.09)	0.02	2.40	0.017

Model 1: Unadjusted

Model 2: Adjusted for sex, age, ethnicity, BMI, education level, marital status, and monthly income.

Model 3: Adjusted for gender, age, ethnicity, BMI, education level, marital status, monthly income, smoking, drinking, physical exercise, diabetes, family history of diabetes, family history of hypertension, and length of employment.

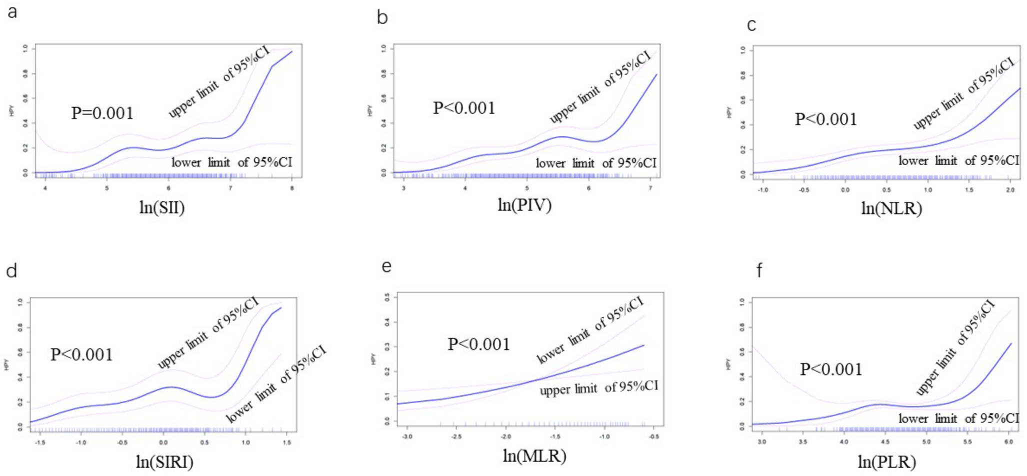

Figure S2. Association between immune-inflammatory indices (ln transformation) and hypertension [a]
SII: Systemic immune inflammatory index; PIV: Pan-immune-inflammation value. SIRI: Systemic inflammatory response index; NLR: Neutrophil-to-lymphocyte ratio; MLR: Monocyte-to-lymphocyte ratio; PLR: Platelet to lymphocyte ratio. All indices were measured at baseline (2013–2015).
a. Adjusted for gender, age, ethnicity, BMI, education level, marital status, monthly income, smoking, drinking, physical exercise, diabetes, family history of diabetes, family history of hypertension, and length of employment.

Table S7 Threshold effect analysis of immune-inflammatory indices (ln transformation) on hypertension using segmented logistic regression.

Outcome	RR((95%CI))	P
ln (SII)		
Standard linear model	1.85(1.50-2.29)	<0.001
Inflection point	4.90	
<4.90	8.19e+129(Inf-Inf)*	0.978
>4.90	1.55(1.18-2.00)	0.002
P for the log-likelihood ratio test	0.001	
ln (PIV)		
Standard linear model	1.86(1.54-2.25)	<0.001
Inflection point	4.01	
<4.01	7.00(2.59-8.19)	0.059
>4.01	1.62(1.29-2.03)	<0.001
P for the log-likelihood ratio test	0.009	
ln (NLR)		
Standard linear model	2.04(1.62-2.57)	<0.001
Inflection point	-0.40	
<-0.40	3.58e+253(5.82e+82-Inf)*	0.979
>-0.40	1.73(1.29-2.29)	<0.001
P for the log-likelihood ratio test	0.005	
ln (SIRI)		
Standard linear model	2.04(1.65-2.50)	<0.001
Inflection point	-1.24	
<-1.24	7.91(3.13-8.19)	0.108
>-1.24	1.74(1.33-2.22)	<0.001
P for the log-likelihood ratio test	0.006	
ln (MLR)		
Standard linear model	1.84(1.27-2.59)	0.002
Inflection point	-1.83	
<-1.83	3.25(1.27-5.97)	0.024
>-1.83	1.40(0.76-2.38)	0.262
P for the log-likelihood ratio test	0.188	
ln (PLR)		
Standard linear model	1.34(0.90-1.95)	0.143
Inflection point	5.20	
<5.20	1.13(0.73-1.73)	0.575
>5.20	5.20(1.14-7.78)	0.036
P for the log-likelihood ratio test	0.066	

* The model failed because of the small sample size.

Adjusted for gender, age, ethnicity, BMI, education level, marital status, monthly income, smoking, drinking, physical exercise, diabetes, family history of diabetes, family history of hypertension, and length of employment.

Table S8

Correlation Analysis						
	ln (SII)	ln (PIV)	ln (NLR)	ln (SIRI)	ln (MLR)	ln (PLR)
ln (SII)	1	.901**	.946**	.845**	.207**	.487**
ln (PIV)	.901**	1	.844**	.957**	.493**	.337**
ln (NLR)	.946**	.844**	1	.884**	.227**	.311**
ln (SIRI)	.845**	.957**	.884**	1	.532**	.168**
ln (MLR)	.207**	.493**	.227**	.532**	1	.325**
ln (PLR)	.487**	.337**	.311**	.168**	.325**	1

The correlation is significant at the 0.01 level.

Table S9 Variance inflation factor (VIF) analysis of four immune inflammation index (ln transformation)

Variable	ln (SII)	ln (PIV)	ln (NLR)	ln (SIRI)
VIF	5928.48	3323.63	2291.38	2138.78

Note: A variance inflation factor (VIF) > 10 indicates severe multicollinearity between variables; all VIF values of the indices in this table are much greater than 10, suggesting the presence of severe multicollinearity.

Table S10 Immune inflammation index (ln transformation) factor analysis

Immune inflammation index (ln transformation)	MR1	h2	Variance explained (%)
ln (SII)	0.95	0.90	90
ln (PIV)	0.95	0.91	
ln (NLR)	0.94	0.88	
ln (SIRI)	0.95	0.89	

Note: MR1 > 0.7 are significant.

The factor analysis results revealed that the extracted "comprehensive inflammatory index" accounted for 90.0% of the cumulative variance. All factor loadings for the included indicators exceeded 0.94 (range: 0.94–0.95), with commonality values ranging from 0.88 to 0.91 (Table S3). This indicates that the common factor adequately reflects the core immune-inflammatory information of the original indicators.
